# Mapping social work scholarship in child and adolescent mental health: a bibliometric analysis of Web of Science and Scopus, 2000–2025

**DOI:** 10.3389/fped.2026.1936049

**Published:** 2026-09-10

**Authors:** Osman Akay

**Affiliations:** 1 Department of Social Work, Seydikemer School of Applied Sciences, Muğla Sıtkı Koçman University, Muğla, Türkiye; 2 Department of Social Work, Faculty of Health Sciences, Istanbul Medipol University, Istanbul, Türkiye

**Keywords:** bibliometrics, child and adolescent mental health, child welfare, health equity, school mental health, social pediatrics, social work, trauma-informed care

## Abstract

**Background:**

Child and adolescent mental health care depends on coordination among pediatric services, mental health care, schools, child welfare, family support, and community systems. Social work occupies a boundary-spanning position in this landscape, yet the organization and development of the relevant scholarship remain unclear.

**Methods:**

Records published between 2000 and 2025 were retrieved from the Web of Science Core Collection and Scopus. The search captured literature explicitly identified as psychiatric, mental health, or clinical social work together with broader social work scholarship situated in child and adolescent mental health. Records were merged, deduplicated, screened for relevance, and analyzed with bibliometrix in R and VOSviewer. The focused analysis examined temporal growth, publication venues, international collaboration, cited-author co-citation, author-keyword co-occurrence, thematic structure, and interperiod thematic flows.

**Results:**

The final corpus comprised 2,623 publications. Annual output increased from 35 publications in 2000 to 254 in 2025; the compound annual growth rate was 8.3%, with a similar estimate when 2024 was used as the endpoint. The literature appeared in 837 sources. Country-affiliation data were available for 2,594 publications; the United States, United Kingdom, Canada, and Australia were the most frequently represented countries, and 14.6% of country-identified publications involved more than one country. Co-citation patterns indicated five intersecting traditions: developmental and ecological theory; trauma and resilience; evidence-based service implementation; social work practice knowledge; and workforce well-being. Six keyword domains connected service systems and implementation, clinical and family-centered practice, child protection, trauma and resilience, youth mental health and treatment, and social determinants affecting marginalized populations. Mental health and social work showed the strongest thematic continuity. Trauma, adverse childhood experiences, resilience, family support, and service-system concerns became more visible in recent periods. Integrated pediatric and primary care, digital mental health, and scholarship from low- and middle-income settings were comparatively less prominent.

**Conclusion:**

The field has expanded beyond individual clinical care, but its knowledge base remains geographically uneven and is more developed in child welfare, school, trauma, and family-service contexts than in integrated pediatric care. Future research should test social work contributions to cross-sector integration, access, continuity, family experience, and health equity.

## 1 Introduction

Mental health conditions commonly emerge during childhood and adolescence, yet access to timely, developmentally appropriate care remains limited in many settings. Current international guidance treats child and adolescent mental health as a shared responsibility across health, education, social care, community services, and justice systems rather than as the exclusive domain of specialist psychiatry (1). This position reflects a broader recognition that emotional and behavioral difficulties are shaped by family resources, school environments, exposure to adversity, neighborhood conditions, poverty, discrimination, displacement, and the organization of services (2–6). The practical consequence is clear: effective care requires more than symptom-focused treatment. It depends on accessible entry points, continuity across settings, family involvement, safeguarding, and coordinated responses to social needs.

Social work is situated at the intersection of these systems. In pediatric and child mental health contexts, social workers conduct psychosocial assessment, support children and caregivers, coordinate care, address barriers to access, contribute to safeguarding, connect families with social resources, and help multidisciplinary teams interpret the social conditions surrounding a child's health. Their contribution is especially visible where mental health concerns overlap with child protection, school participation, housing instability, family stress, disability, substance use, or involvement with multiple agencies. Ecological theory provides a durable conceptual basis for this work by locating child development within interacting family, institutional, community, and societal environments (7). Service research has likewise emphasized the importance of evidence-based practice, school-based care, integrated behavioral health, and implementation processes that connect interventions with the settings in which children live and receive support (8–12).

The need for cross-sector care has become more explicit. A recent systematic review found that integrated youth mental health models can improve several clinical and service outcomes, although effects vary across models and contexts (13). Evidence on integrated health and social care for underserved children also points to promising forms of targeted, collaborative, and school-centered support, while noting inconsistent evaluation and persistent uncertainty about the contribution of social care within integrated teams (14). Evidence from a Quebec child welfare sample indicates that young people may use several service sectors, highlighting coordination needs that isolated referrals are unlikely to meet (15). These findings place social work within a broader pediatric service question: how can care systems respond to mental health needs that are inseparable from family, educational, protective, and structural conditions?

For pediatric practice and service organization, this question is not limited to the presence of social workers within hospitals or clinics. It concerns how pediatric services connect with schools, child welfare, family services, and community mental health; which social work roles are visible at these interfaces; and where coordination-related knowledge remains limited. Mapping this scholarship can therefore show which pediatric–social service interfaces have received sustained attention and identify priorities for evaluative research on referral pathways, family support, care continuity, and integrated behavioral health. It does not determine whether particular pediatric service models are effective.

The scholarship addressing this question is dispersed across social work, psychiatry, psychology, pediatrics, public health, education, and child welfare journals. Its boundaries are not fully captured by the phrase “psychiatric social work”. Many relevant studies use broader terms such as social work, social services, child welfare, school social work, clinical social work, or integrated care. This terminological spread creates a fragmented evidence base. It also complicates attempts to determine which service domains have shaped the field, whether the literature has moved toward more integrated and preventive orientations, and which populations or regions remain weakly represented.

In this study, social work scholarship is treated as an operational, record-level category rather than being inferred from disciplinary affiliation or publication venue. It includes records whose titles, abstracts, or keywords demonstrate substantive attention to social workers or the social work workforce; social work assessment, practice, intervention, case management, or care coordination; or social-work-related service and policy processes within child and adolescent mental health. Publication in a social work journal, an author's social work affiliation, or an isolated occurrence of social work terminology was not sufficient for inclusion. Scholarship from adjacent service domains was considered only when the available metadata indicated both a child or adolescent mental health component and an explicit social work role or process.

Direct psychiatric, clinical, or mental health social work places social workers, social work practice, or the profession's workforce explicitly within mental health assessment, treatment, or service delivery. The adjacent domains represented in this review are organized around different institutional settings, including child welfare and protection, schools, foster or kinship care, pediatric integrated care, and family or community services. These domains were examined within the same corpus because children's mental health needs often extend across service settings and because social workers connect these settings through psychosocial assessment, safeguarding, referral, care coordination, family support, and responses to trauma and social adversity. Their inclusion does not mean that all child-service research was treated as social work scholarship; only records demonstrating a substantive connection among social work, a child or adolescent population, and mental health were eligible.

Earlier bibliometric studies have mapped child and adolescent mental health intervention research, reporting increasing publication output, Western geographic concentration, and growing interdisciplinary collaboration (16). Other mappings have focused on more specific segments, including child and adolescent mental health during the COVID-19 pandemic (17), global child maltreatment research (18), and social work scholarship produced in response to COVID-19 (19). More recent work has examined child abuse and neglect explicitly through a social work lens centered on child welfare and child protection systems (20). Taken together, these studies provide valuable accounts of publication growth, geographic distribution, collaboration, and topic-specific knowledge structures. Their analytical boundaries, however, are generally defined by broad intervention research, a particular public health context, or a specific problem domain such as child maltreatment. The wider knowledge structure formed by social work across child and adolescent mental health, pediatric care, child welfare, schools, and family or community services remains insufficiently examined. This study extends the existing mapping literature by applying a record-level operational definition and examining intellectual, conceptual, temporal, and geographic relationships across these service interfaces.

Bibliometric analysis is useful when a field is large, interdisciplinary, and conceptually distributed. Performance indicators describe growth and concentration, whereas science-mapping techniques reveal relationships among authors, cited traditions, documents, and concepts (21–24). These methods do not evaluate intervention effectiveness or study quality. Their value lies in showing how a body of scholarship is organized, where it is concentrated, and how its conceptual priorities change. In the present study, the novelty does not rest on combining two databases or applying a large number of standard techniques. It lies in interpreting the literature as a knowledge structure at the interface of child mental health, pediatrics, child welfare, schools, family services, and community care.

The study contributes an operationally bounded, cross-database account of a literature that has previously been dispersed across psychiatric social work, child welfare, school mental health, family services, pediatrics, and community care. By bringing performance indicators, co-citation patterns, keyword structure, and thematic change into a single interpretive framework, it shows not only where publications accumulate but also how the field's intellectual traditions and service orientations are connected and where important gaps remain. This integrated mapping provides a field-level basis for setting research priorities at the interface of social work and child and adolescent mental health services.

This study therefore maps social work scholarship in child and adolescent mental health between 2000 and 2025. Four questions guide the analysis: (1) How has the literature developed in volume, publication location, and international collaboration? (2) Which intellectual traditions and service domains organize the field? (3) How have its conceptual priorities changed across time? (4) What forms of geographic and service-system concentration or underrepresentation are visible in the mapped literature?

## 2 Materials and methods

### 2.1 Study design and reporting framework

This study used bibliometric performance analysis and science mapping to examine the development, intellectual organization, conceptual structure, and international distribution of social work scholarship related to child and adolescent mental health. Reporting was informed by the BIBLIO guidance for bibliometric reviews, PRISMA-S for transparent reporting of literature searches, and relevant elements of PRISMA 2020 for record identification and selection (25–27). The flow diagram was adapted to the construction of a bibliometric corpus; no risk-of-bias assessment was undertaken because the study did not synthesize intervention effects.

The analytical scope was limited to English-language journal articles and reviews published between 2000 and 2025. No geographic restriction was applied; records from all countries represented in the eligible Web of Science and Scopus results were considered. Books, book chapters, conference papers, editorials, letters, notes, dissertations, gray literature, and other non-article formats were outside the scope of the corpus. Accordingly, the term social work scholarship in this study refers specifically to the eligible journal literature captured by the stated databases, period, language, publication-type criteria, and substantive relevance requirements.

### 2.2 Information sources and search dates

Records were retrieved from the Web of Science Core Collection and Scopus. These databases were selected because they provide structured metadata required for citation analysis, co-authorship mapping, keyword analysis, and cited-reference networks. The Scopus search was conducted on 23 December 2025. The Web of Science search was completed on 24 December 2025. Fixed search dates were used because database indexing and citation counts change over time.

Web of Science records were exported in BibTeX format with Full Record and Cited References. Scopus records were exported in CSV format with complete bibliographic information and references when available. The study period was restricted to 2000–2025. Publications from late 2025 may be affected by indexing delay; a sensitivity calculation using 2024 as the endpoint was therefore included for the annual growth estimate.

### 2.3 Search strategy and conceptual scope

The search was designed to identify literature at the intersection of social work, mental health, and child or adolescent populations. The Web of Science Topic search was:

((“psychiatric social work” OR “mental health social work” OR “clinical social work”) OR (“social work” AND (psychiatr* OR “mental health”))) AND (child* OR adolescen* OR youth OR pediatric*)

The Scopus search used the corresponding TITLE-ABS-KEY syntax:

TITLE-ABS-KEY (((“psychiatric social work” OR “mental health social work” OR “clinical social work”) OR (“social work” AND (psychiatr* OR “mental health”))) AND (child* OR adolescen* OR youth OR pediatric*))

The strategy intentionally extended beyond publications that explicitly used the label “psychiatric social work”. This decision reflected variation in disciplinary terminology and the fact that clinically and systemically relevant social work research is frequently indexed under child welfare, school social work, social services, psychosocial care, or mental health services. The resulting corpus is therefore interpreted as social work scholarship situated within child and adolescent mental health, not as a closed set of publications belonging to a single formally bounded specialty.

In Web of Science, eligible records were restricted to Article, Review Article, and Early Access; English language; publication years 2000–2025; and the Social Sciences Citation Index, Science Citation Index Expanded, or Emerging Sources Citation Index. The Web of Science search yielded 1,438 records. The unfiltered Scopus search returned 4,110 records. After year, language, source-type, and document-type limits were applied, 2,203 Scopus records remained.

### 2.4 Eligibility criteria

Eligibility required all four of the following conditions: (1) an eligible publication type, language, and publication year; (2) a central or analytically separable child or adolescent population or service context; (3) a substantive mental health component; and (4) a substantive social work component. A mental health component was considered substantive when the record addressed emotional, behavioral, or psychiatric conditions, psychosocial assessment or intervention, mental health service use, or the organization and delivery of mental health-related care. A social work component was considered substantive when the title, abstract, or keywords referred to social workers or the social work workforce; social work assessment, practice, intervention, case management, or care coordination; or service and policy processes explicitly involving social work.

Psychiatric or clinical services, child welfare, child protection, school mental health, family services, foster care, trauma-related services, integrated care, and community support were treated as settings in which eligible social work scholarship could occur rather than as independent grounds for inclusion. Records from these adjacent domains were included only when the available metadata established a substantive connection among social work, a child or adolescent population, and mental health. General studies of these service settings without such a connection were excluded. Publication in a social work journal, an author's social work affiliation, or an isolated occurrence of social work terminology was not sufficient for eligibility.

Conference papers, book chapters, editorials, letters, notes, publisher-invited open reviews, and other non-article formats were excluded. Records were also excluded when their substantive focus was adult-only mental health, physical health without a meaningful psychosocial or mental health component, nutrition, general medical cost analysis, perinatal or postpartum care without child or adolescent mental health relevance, or professional education without a direct connection to child or adolescent mental health services.

Operationally, social work scholarship was defined at the record level. Eligible records were required to contain a substantive social work component, reflected in explicit attention to social workers or the social work workforce; social work practice, assessment, intervention, case management, or care coordination; or social-work-related service and policy processes. Publication in a social work journal, a social work author affiliation, or an incidental reference to social work was not considered sufficient. Studies situated in child welfare, child protection, foster care, school mental health, family services, integrated care, or community support were included only when the available metadata also demonstrated both a child or adolescent mental health focus and an explicit social work role or process.

### 2.5 Data integration, deduplication, and relevance screening

The two database exports were imported into R and converted to a common bibliographic structure using bibliometrix (21). A source-database field and the original Web of Science and Scopus identifiers were retained throughout the process. Deduplication proceeded in two stages. First, DOI values were converted to lowercase, trimmed, and standardized. Records sharing a DOI were merged, resulting in the removal of 821 redundant records. Second, titles were converted to lowercase, stripped of punctuation, and normalized for spacing. Potential title matches were manually compared before merging. Eleven additional redundant records were identified through this procedure.

Duplicate records were reconciled at the field level rather than by discarding all metadata from one database version. Core bibliographic fields were taken from the Web of Science record when available, and missing fields were completed from the corresponding Scopus record. When both databases supplied an abstract, the more complete version was retained. Author keywords and index keywords were pooled across database versions and deduplicated case-insensitively. Web of Science and Scopus affiliation strings were retained in source-specific fields, while cited references were pooled and deduplicated using DOI values when available and normalized reference strings otherwise. Database-specific citation counts and record identifiers were retained separately and were not summed. This procedure preserved complementary metadata while maintaining one analytical record for each duplicated publication.

**Figure 1:**
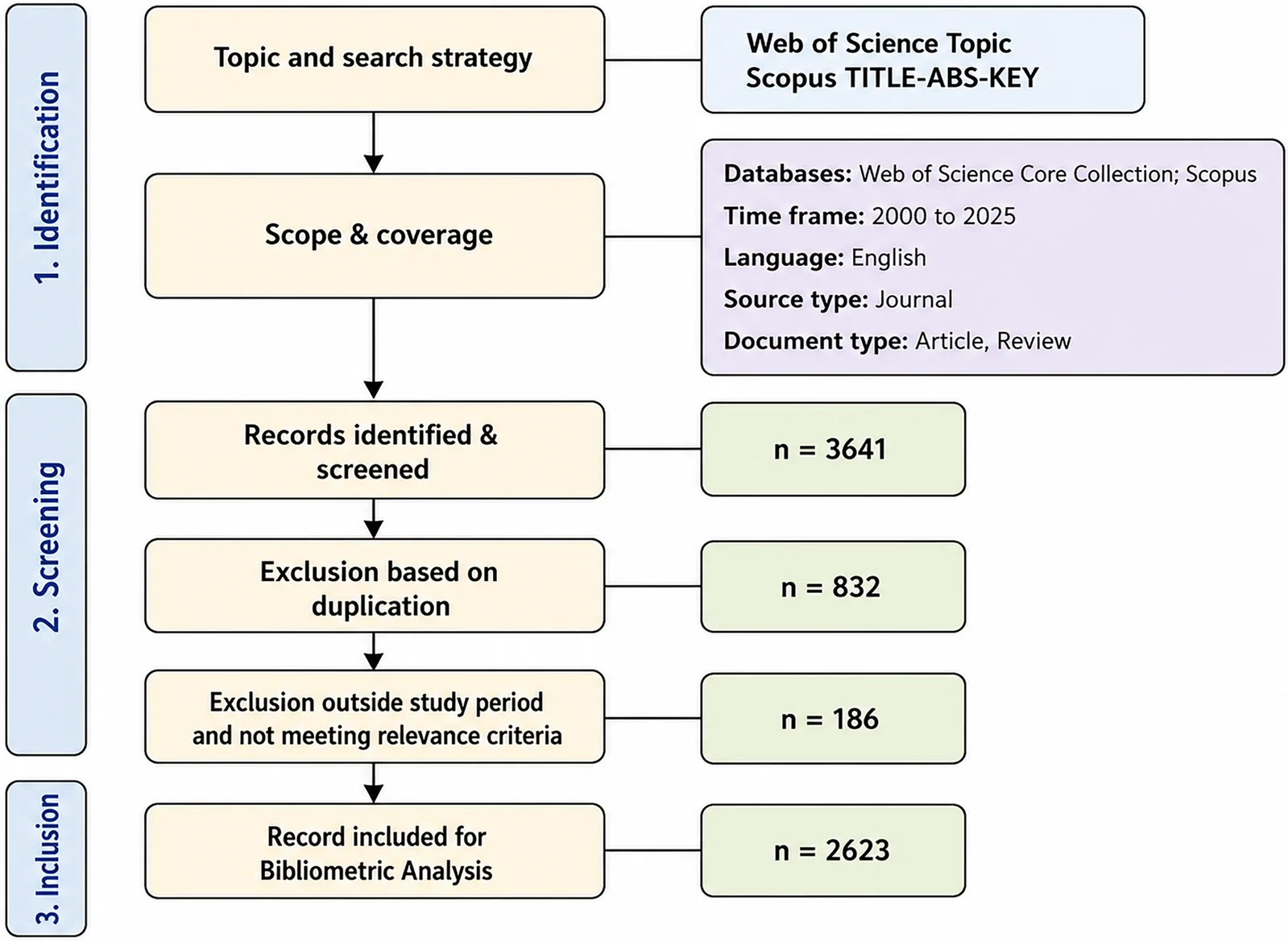
Flow diagram for bibliometric corpus construction. The diagram distinguishes duplicate removal, exclusion of records outside the study period, relevance screening, and final inclusion.

The merged dataset contained 3,641 records before deduplication and 2,809 unique records afterward. Two records indexed as 2026 publications were removed. The remaining 2,807 records were screened using titles, abstracts, author keywords, and index keywords. Screening applied the predefined conceptual and eligibility criteria described above. A total of 184 records were excluded, leaving 2,623 publications in the final corpus (Figure 1).

Relevance screening was conducted by one reviewer using titles, abstracts, author keywords, and index keywords. Decisions followed four sequential criteria: (1) eligible publication characteristics; (2) a central or analytically separable child or adolescent population or service context; (3) a substantive mental health component; and (4) a substantive social work component as defined above. When the title alone was insufficient, the abstract and both keyword fields were examined together. Ambiguous records were set aside and re-examined across all available metadata fields before a final decision was made. Exclusion was applied only when at least one predefined criterion was clearly unmet. Records whose relevance could not be ruled out on metadata grounds were retained to prioritize recall. Screening was conducted at the metadata level and was not independently duplicated.

### 2.6 Data cleaning and harmonization

Quality checks examined missing titles, publication years outside the study period, inconsistent document types, incomplete affiliations, and source-title anomalies. Before keyword-network construction, author keywords were converted to lowercase, trimmed, and standardized for punctuation, hyphenation, singular and plural forms, and British and American spelling variants. A thesaurus was then applied to abbreviations and lexical variants representing the same substantive concept. Examples included “children” to “child,” “adolescents” to “adolescent,” “social workers” to “social worker,” “paediatric” to “pediatric,” “behavioural health” to “behavioral health,” and “PTSD” to “post-traumatic stress disorder”.

Terms were merged only when semantic equivalence could be established independently of their position in the network. Conceptually related but non-equivalent terms were retained separately. These included “child welfare” and “child protection,” “child abuse” and “child maltreatment,” “trauma” and “adverse childhood experiences,” “social work” and “social services,” “mental health” and “psychiatry,” and “family support” and “social support”. Population terms such as “child,” “adolescent,” “youth,” and “young people” were also retained separately because they may represent different age or service categories. Likewise, “clinical social work,” “psychiatric social work,” and “school social work” were not collapsed into a single term because their service settings are analytically relevant. Representative merged terms and deliberately retained distinctions are reported in Supplementary Table S1.

Institutional metadata were not used for substantive interpretation in the main analysis because raw affiliation fields mixed university systems, individual universities, faculties, departments, and service organizations. This decision avoided comparisons across non-equivalent organizational levels. Institution-level results may be reported only after a separate affiliation-normalization procedure.

### 2.7 Bibliometric analysis

Analyses were conducted in R with bibliometrix and in VOSviewer version 1.6.20 (21, 22). The study retained a focused set of analyses aligned with the research questions.

Temporal development was examined through annual publication counts, period-specific totals, mean annual output, and compound annual growth rate. A sensitivity calculation ending in 2024 assessed whether the growth estimate was driven by the final year. Publication ecology was described through source productivity and dispersion. International structure was assessed through country-level output and country co-authorship.

Country-level analyses used the publication as the unit of analysis. Country names were extracted from all available author affiliations and standardized before counting. Repeated occurrences of the same country within a publication, whether arising from multiple authors or multiple affiliations, were counted once. Country output was calculated using full counting: each country represented in a publication received one publication credit. Consequently, a publication involving more than one country contributed one credit to each participating country, and country shares were non-exclusive. Percentages were calculated using the 2,594 publications with available country information as the denominator. Publications were classified as internationally collaborative when their affiliation metadata contained at least two distinct countries; 29 records without usable country information were excluded from this calculation. The country co-authorship network also used full counting. A link represented joint participation in at least one publication, and total link strength represented the sum of a country's co-authorship link weights.

Cited-author co-citation analysis was used to identify the intellectual traditions that were jointly referenced within the corpus. Co-citation links authors who are cited together and is suited to mapping a field's knowledge base (28). Author-keyword co-occurrence analysis was used to identify conceptual domains. Thematic mapping positioned themes by centrality and density, while thematic evolution and the weighted inclusion index were used to examine continuity between consecutive periods. The periods 2000–2012, 2013–2019, and 2020–2025 were selected to distinguish an early phase, a period of consolidation, and a recent phase marked by accelerated publication growth and the COVID-19 pandemic.

VOSviewer networks used full counting and association-strength normalization. Thresholds were set to preserve a sufficiently broad network while maintaining label legibility in a corpus of this size: five documents for countries, 30 citations for cited authors, and 20 occurrences for author keywords. These cutoffs retained 40 countries, 65 cited authors, and 57 keywords. No formal threshold-sensitivity analysis was conducted; low-frequency items were therefore interpreted as less visible within the selected maps rather than absent from the literature. Cluster labeling and thematic interpretation were conducted by the author after the VOSviewer clustering solutions had been generated. Cluster membership remained algorithmically determined, and no nodes were reassigned to improve thematic coherence. For each cluster, the complete membership list was reviewed rather than only the largest or most visible nodes. Provisional labels were developed from multiple nodes with the highest citation frequency, keyword occurrence, or total link strength, as appropriate to the network. Lower-weight and potentially discordant nodes were then examined to determine whether the provisional label adequately represented the breadth of the cluster. When a cluster contained more than one intellectual tradition or service area, the label was broadened and the overlap was acknowledged in its interpretation. Ambiguous nodes were examined within their bibliographic or keyword context before the final label was selected. The resulting labels are interpretive, non-exclusive summaries of algorithmically generated clusters rather than objective or pre-specified categories.

The focused analysis prioritized outputs that directly addressed the four research questions. Bradford's Law, Lotka's Law, author productivity rankings, author co-authorship, institutional networks, and document-level bibliographic coupling were not included because they added descriptive detail without strengthening interpretation of the field's service domains, intellectual traditions, or knowledge inequalities.

### 2.8 Interpretive approach

Bibliometric structures were interpreted against contemporary scholarship on child and adolescent mental health services, social determinants, child welfare, school mental health, trauma-informed care, and integrated health and social care. Three principles guided interpretation. Publication volume was not equated with scientific quality or service effectiveness; citation prominence was treated as influence within the indexed corpus rather than proof of conceptual validity; and absence from a thresholded map was read as low visibility among high-frequency items, not as evidence that a topic was missing from the literature. Ecological theory served as an orienting lens rather than an *a priori* coding framework. Its later prominence in the co-citation network was consequently treated as a feature of the corpus itself, not as a category introduced by the analysts.

## 3 Results

### 3.1 Corpus construction and temporal development

The searches identified 1,438 Web of Science records and 2,203 Scopus records after database filters, yielding 3,641 records before deduplication. DOI matching removed 821 records and normalized-title matching removed a further 11. Two records outside the predefined period and 184 records that did not meet the relevance criteria were excluded. The final corpus comprised 2,623 publications (Figure 1; Table 1).

**Table 1:** Corpus construction and descriptive overview.

Indicator	Value
Web of Science records after database limits	1,438
Scopus records after database limits	2,203
Records before deduplication	3,641
DOI duplicates removed	821
Title duplicates removed	11
Records excluded outside 2000–2025	2
Records excluded after relevance screening	184
Final publications	2,623
Publication sources	837
Unique authors	9,060
Publications with country information	2,594
Countries represented	84
Multi-country publications	380 (14.6%)
Compound annual growth rate, 2000–2025	8.3%
Sensitivity growth rate, 2000–2024	8.1%

The percentage of multi-country publications was calculated using the 2,594 publications with available country information as the denominator.

**Figure 2:**
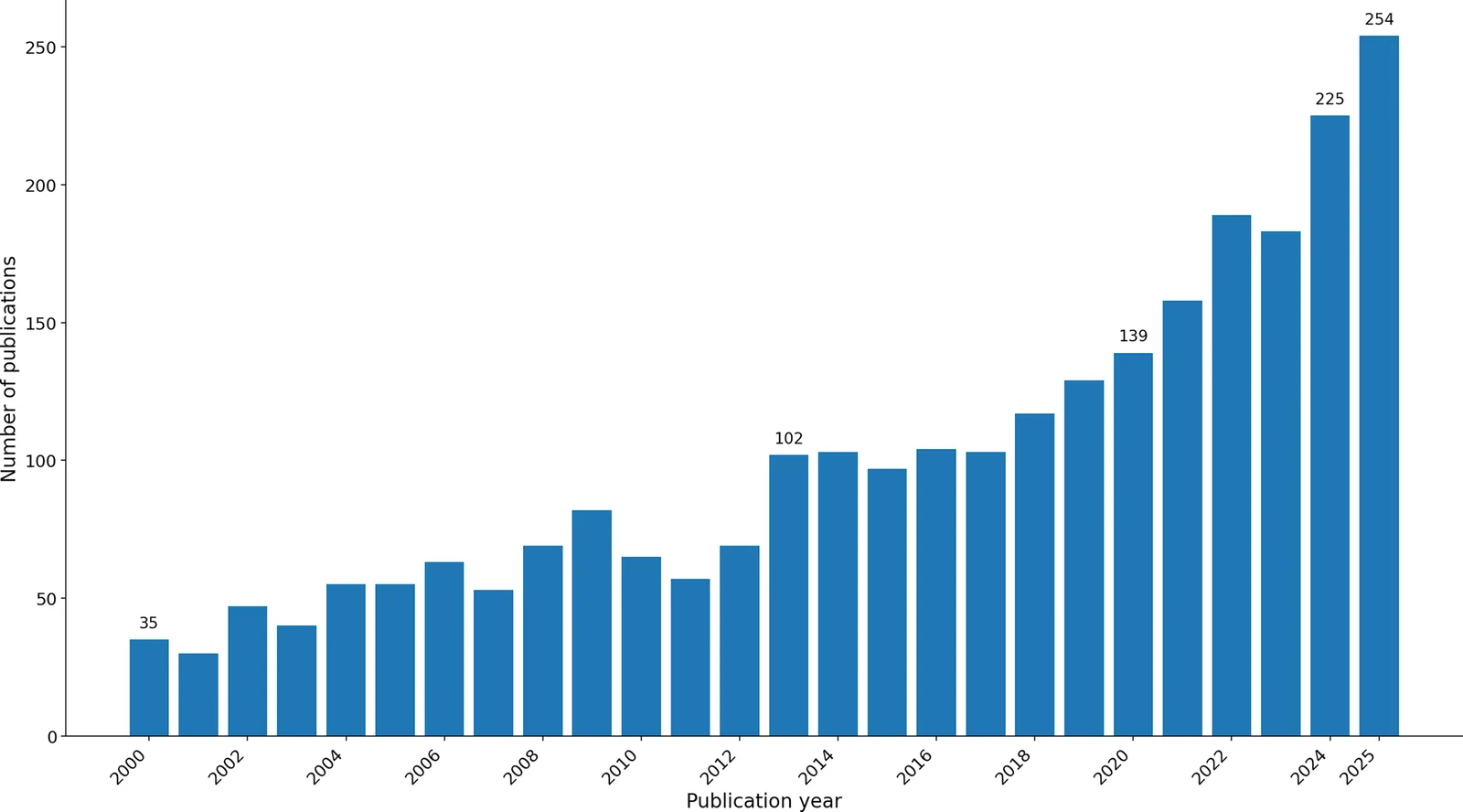
Annual publication output in social work scholarship related to child and adolescent mental health, 2000–2025. The final year may be affected by database indexing delay; the 2000–2024 sensitivity calculation yielded a similar growth estimate.

Annual publication output increased from 35 records in 2000 to 254 in 2025 (Figure 2). Output remained below 100 publications per year through 2012, exceeded that level in 2013, and rose more consistently after 2017. The period 2021–2025 contributed 1,009 publications, representing 38.5% of the entire corpus. Mean annual output increased from 52.9 publications in 2000–2009 to 201.8 in 2021–2025. The compound annual growth rate was 8.3% for 2000–2025. Using 2024 as the endpoint produced a comparable estimate of 8.1%, indicating that the long-term pattern was not dependent on the 2025 count.

### 3.2 Publication ecology and international concentration

The 2,623 publications appeared in 837 sources. The British Journal of Social Work contributed 70 publications, followed by Clinical Social Work Journal and Journal of Social Work with 53 each. Research on Social Work Practice, Children and Youth Services Review, and Child and Adolescent Social Work Journal each contributed approximately 50 publications (Table 2). The source list included journals categorized within social work and child services as well as journals in psychiatry, psychology, pediatrics, public health, education, and health services.

**Table 2:** Leading publication venues and countries.

Panel A. Publication venues			
Rank	Source	Publications	Total citations
1	British Journal of Social Work	70	974
2	Clinical Social Work Journal	53	766
3	Journal of Social Work	53	623
4	Research on Social Work Practice	52	1,101
5	Children and Youth Services Review	52	1,008
6	Child and Adolescent Social Work Journal	52	598

Panel B. Countries				
Rank	Country	Publications	Share of corpus	Total link strength
1	United States	1,266	48.8%	238
2	United Kingdom	420	16.2%	187
3	Canada	268	10.3%	91
4	Australia	184	7.1%	113
5	China	81	3.1%	72
6	Sweden	75	2.9%	23

Country output was calculated using full counting at the publication level. Each country was counted no more than once within a publication, but internationally co-authored publications contributed one publication credit to each participating country. Country shares therefore represent non-exclusive proportions and may sum to more than 100%. Percentages were calculated using the 2,594 publications with available country information as the denominator. Total link strength was obtained from the full-counting country co-authorship network.

Country information was available for 2,594 of the 2,623 publications. The country-affiliation data represented 84 countries, but participation was concentrated in a small group. Using full counting, the United States was represented in 1,266 publications (48.8% of records with country information), followed by the United Kingdom with 420 (16.2%), Canada with 268 (10.3%), and Australia with 184 (7.1%). China, Sweden, Hong Kong, Israel, South Africa, and the Netherlands formed a second tier with markedly smaller outputs. Because each participating country received one credit in internationally co-authored publications, these shares are non-exclusive and should not be summed. Among publications with country information, 380 (14.6%) involved affiliations from at least two countries.

**Figure 3:**
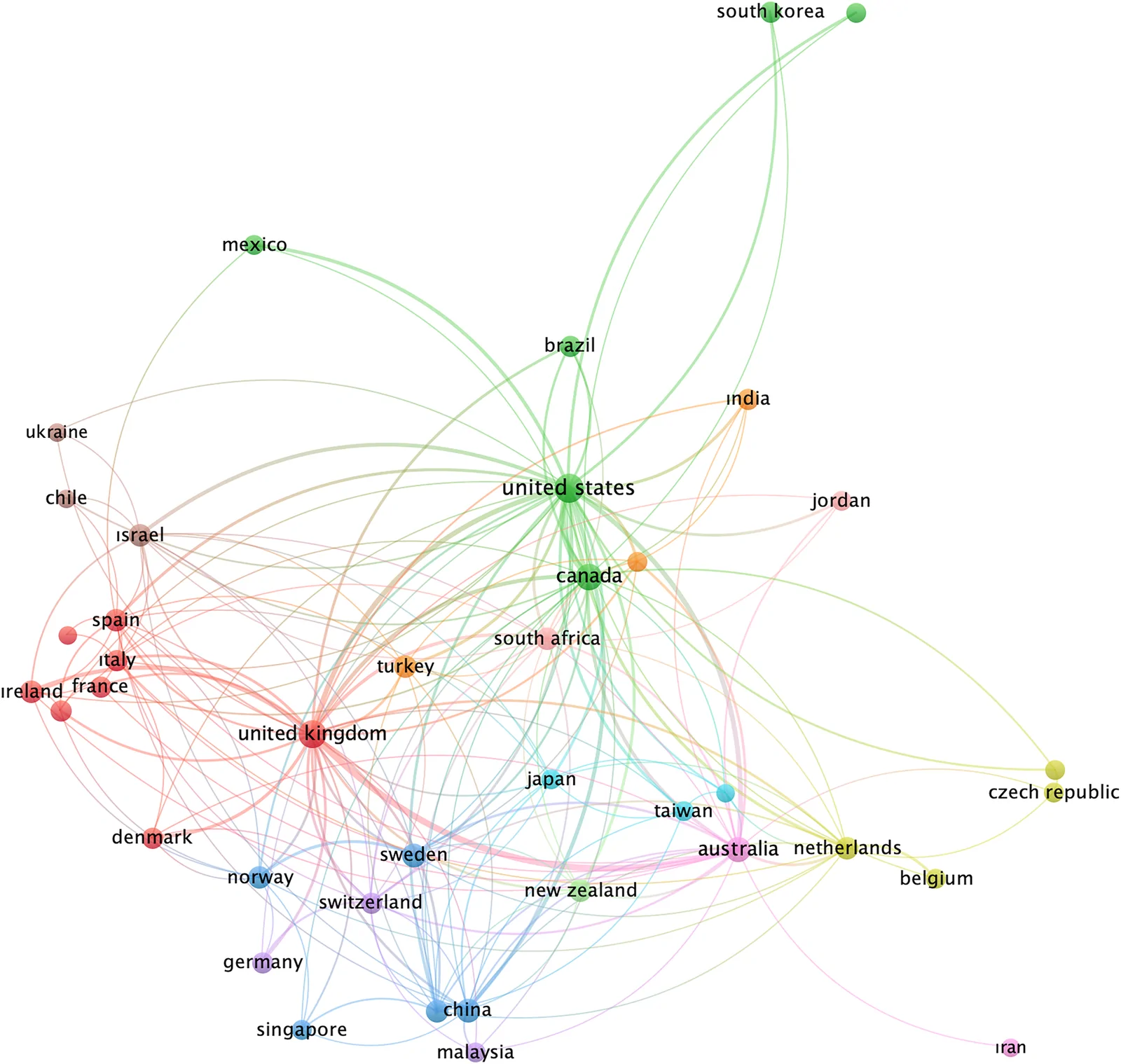
International co-authorship network. Node size indicates the number of publications in which a country was represented, links indicate cross-country co-authorship, link thickness represents link strength, and colors indicate VOSviewer clusters. Full counting was used: each country was counted no more than once within a publication, whereas a publication involving multiple countries contributed one publication credit to each participating country. The network used association-strength normalization and a minimum threshold of five publications per country.

The country co-authorship network contained 40 countries, 185 links, and a total link strength of 579 (Figure 3). The United States had the highest total link strength (238), followed by the United Kingdom (187), Australia (113), Canada (91), and China (72). The United States had co-authorship links with the United Kingdom, Canada, Australia, China, South Africa, Japan, Türkiye, South Korea, Mexico, and several other countries. These measures describe co-authorship relations within the thresholded network and do not measure the organization or effectiveness of international service systems.

### 3.3 Intellectual structure

**Figure 4:**
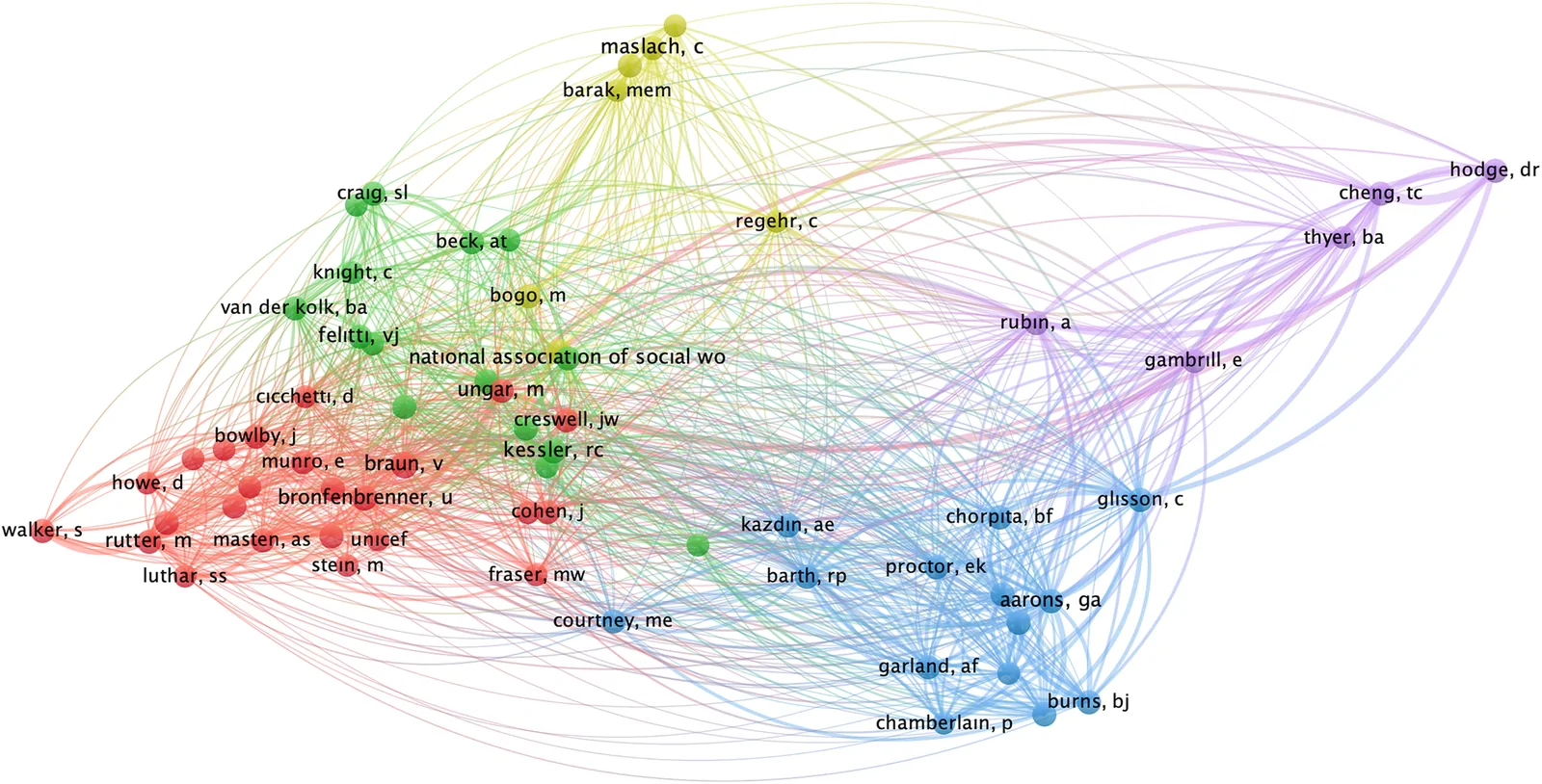
Cited-author co-citation network. Node size indicates citation frequency, links represent co-citation relationships, and colors indicate VOSviewer clusters. The network included 65 cited authors meeting a threshold of 30 citations.

The cited-author co-citation network included 65 authors, five clusters, 995 links, and a total link strength of 7,144 (Figure 4). Following review of complete cluster membership, the five clusters were assigned interpretive labels based on their most prominent and recurrent nodes (Table 3).

**Table 3:** Cited-author co-citation clusters: observed composition and interpretive labels.

Cluster	Interpretive label	Representative high-weight authors	Observed basis for the label
1	Developmental–ecological and resilience scholarship	Bronfenbrenner; Bowlby; Rutter; Masten; Luthar; Munro	Co-citation of authors associated with ecological development, attachment, developmental risk and resilience, and child protection scholarship
2	Trauma and psychosocial assessment	Felitti; van der Kolk; Beck; Ungar; Bogo; Kessler	Co-citation of authors associated with adversity and trauma, clinical assessment, resilience, and psychosocial practice
3	Evidence-based services and implementation	Aarons; Chorpita; Burns; Proctor; Glisson; Garland; Kazdin; Barth	Co-citation of authors associated with intervention evidence, service implementation, organizational context, and public child-serving systems
4	Social work practice knowledge and evidence use	Gambrill; Thyer; Rubin; Hodge; Cheng	Co-citation of authors associated with practice evaluation, professional decision-making, measurement, and evidence use in social work
5	Workforce well-being and organizational strain	Maslach; Barak; Regehr	Co-citation of authors associated with burnout, occupational stress, workforce well-being, and organizational conditions

Cluster membership was determined algorithmically by VOSviewer. Interpretive labels were developed after reviewing the complete membership of each cluster together with the relative prominence of its nodes. The labels describe dominant tendencies rather than every author represented in a cluster. They are non-exclusive summaries and should not be interpreted as objective or mutually exclusive intellectual categories.

The first cluster included Bronfenbrenner, Bowlby, Rutter, Masten, Luthar, and Munro and was labeled developmental–ecological and resilience scholarship. The second included Felitti, van der Kolk, Beck, Ungar, Bogo, and Kessler and was labeled trauma and psychosocial assessment. The third included Aarons, Chorpita, Burns, Proctor, Glisson, Garland, Kazdin, and Barth and was labeled evidence-based services and implementation. The fourth included Gambrill, Thyer, Rubin, Hodge, and Cheng and was labeled social work practice knowledge and evidence use. The fifth included Maslach, Barak, and Regehr and was labeled workforce well-being and organizational strain. These labels summarize dominant co-citation patterns and do not imply that all authors within a cluster represent the same theoretical tradition.

### 3.4 Conceptual domains

**Figure 5:**
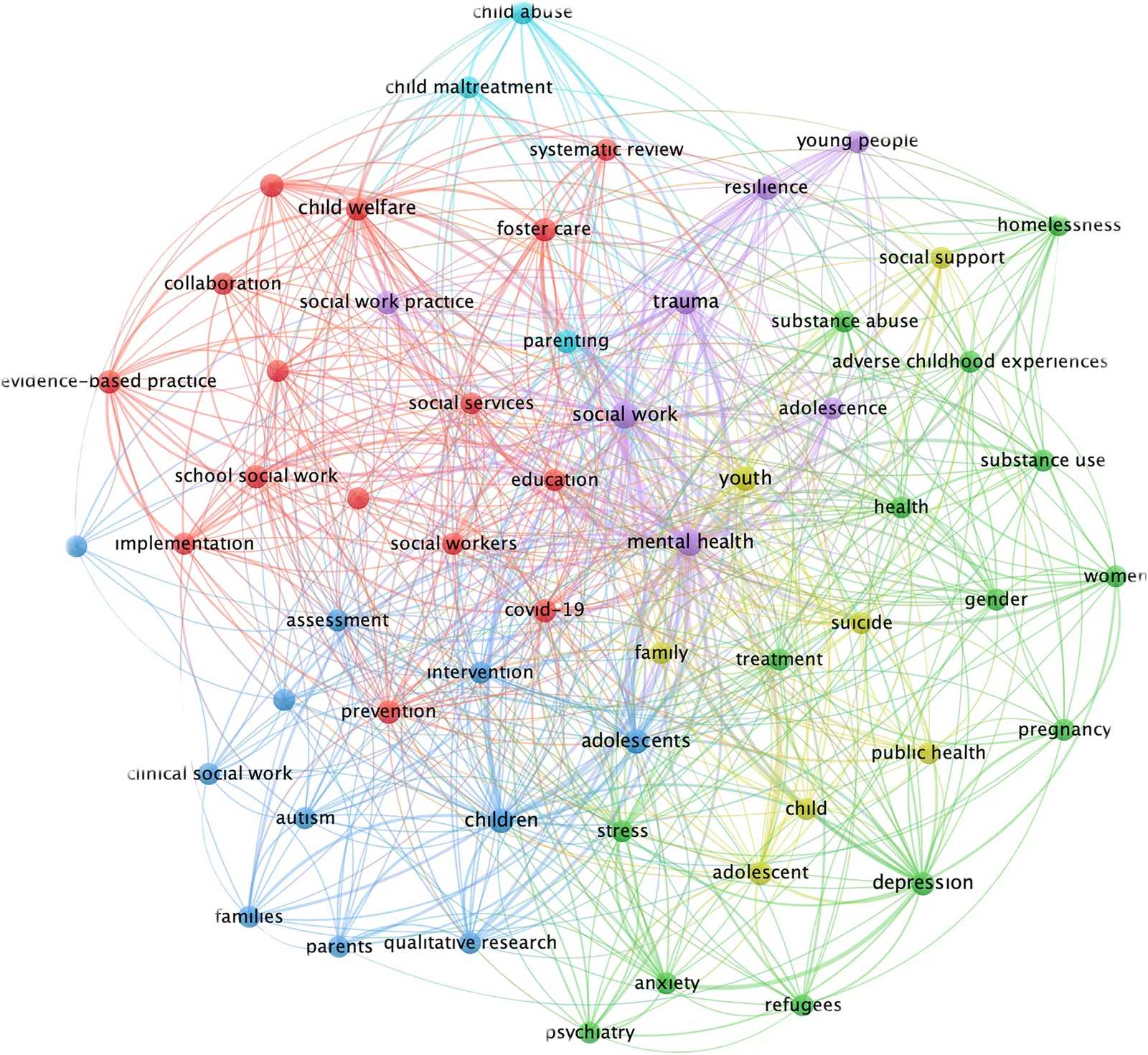
Author-keyword co-occurrence network. Node size indicates keyword frequency, links represent co-occurrence, and colors indicate VOSviewer clusters. The network included 57 author keywords meeting a minimum threshold of 20 occurrences.

The author-keyword network included 57 terms, six clusters, 640 links, and a total link strength of 1,722 (Figure 5). Following review of complete cluster membership, the clusters were assigned the interpretive labels service systems and implementation; clinical and family practice; child protection and maltreatment; trauma, resilience, and adaptation; youth mental health and treatment; and psychiatric and social vulnerability (Table 4).

**Table 4:** Author-keyword clusters: observed composition and interpretive labels.

Cluster	Interpretive label	Representative high-occurrence keywords	Observed basis for the label
1	Service systems and implementation	child welfare; social services; school social work; collaboration; implementation; evidence-based practice; prevention	Co-occurrence of terms concerning organizational settings, interagency activity, implementation, and professional service delivery
2	Clinical and family practice	clinical social work; families; parents; assessment; intervention; autism; children; adolescents	Co-occurrence of terms concerning direct practice, assessment, intervention, and work with children and families
3	Child protection and maltreatment	child abuse; child maltreatment; parenting; foster care	Co-occurrence of terms concerning maltreatment, protection, caregiving, and out-of-home care
4	Trauma, resilience, and adaptation	trauma; resilience; young people; social work practice; systematic review	Co-occurrence of terms concerning adversity, adaptation, resilience, and professional response
5	Youth mental health and treatment	mental health; youth; family; treatment; social support; suicide; adolescent; child	Co-occurrence of terms concerning mental health needs, treatment, family context, support, and acute risk
6	Psychiatric and social vulnerability	depression; anxiety; psychiatry; public health; substance use; homelessness; refugees; gender; pregnancy; adverse childhood experiences	Heterogeneous co-occurrence of psychiatric, public health, adversity, and socially marginalized population terms

Cluster membership was determined algorithmically by VOSviewer. Interpretive labels were assigned after reviewing complete cluster membership, keyword occurrence, and link strength. Labels describe the dominant pattern within each cluster but do not encompass every term or imply conceptual equivalence among all cluster members. Related themes may overlap across clusters.

The first cluster included child welfare, social services, school social work, collaboration, implementation, evidence-based practice, prevention, education and social workers. The second included clinical social work, families, parents, assessment, intervention, autism, children, adolescents, and qualitative research. The third included child abuse, child maltreatment, parenting, foster care, and related protection terms. The fourth included trauma, resilience, young people, social work practice, and systematic review. The fifth included mental health, youth, family, treatment, social support, suicide, adolescent, and child. The sixth cluster included depression, anxiety, psychiatry, public health, substance use, homelessness, refugees, gender, women, pregnancy, and adverse childhood experiences. Because these terms represented several related but non-equivalent strands, the cluster was given the broad interpretive label psychiatric and social vulnerability. The labels assigned to these clusters are interpretive summaries of term co-occurrence and do not represent objective or mutually exclusive disciplinary categories.

In the thematic map, mental health, child welfare, depression, and family support were positioned as basic themes; community was positioned as a motor theme; school mental health and quality improvement were classified as specialized themes; and sexual abuse was located in the emerging-or-declining quadrant. Primary care, integrated pediatric care, digital mental health, telehealth, and formal health–social care integration were not among the keywords meeting the minimum threshold of 20 occurrences. Their absence from this thresholded map indicates lower visibility among high-frequency author keywords and does not establish that these topics were absent from the corpus.

### 3.5 Thematic development across periods

Thematic evolution showed strong continuity in the core vocabulary of the field (Table 5). The most substantial flow linked mental health in 2013–2019 with mental health in 2020–2025 (weighted inclusion index = 0.584). Shared terms included youth, stigma, adolescence, empowerment, intellectual disability, quality of life, pediatrics, and substance use. The second strongest flow connected social work across the same periods (0.420), with child protection, social workers, culture, social justice, and children and families among the shared terms.

**Table 5:** Strongest interperiod thematic flows.

Previous-period theme	Subsequent-period theme	Shared terms (selected)	Weighted inclusion index
mental health (2013–2019)	mental health (2020–2025)	mental health; youth; stigma; adolescence; empowerment; intellectual disability; quality of life; pediatrics; substance use	0.584
social work (2013–2019)	social work (2020–2025)	social work; child protection; social workers; culture; social justice; children and families	0.420
children (2000–2012)	children (2013–2019)	children; adolescents; school social work; attachment; schools	0.318
mental health (2000–2012)	mental health (2013–2019)	mental health; substance abuse; women	0.318
health (2013–2019)	mental health (2020–2025)	interventions; treatment; alcohol; epidemiology; mental disorders	0.286

Additional interperiod links connected children in 2000–2012 with children in 2013–2019 and mental health in 2000–2012 with mental health in 2013–2019 (Table 5). The corresponding weighted inclusion indices are reported in Table 5 together with the shared terms supporting each flow.

In the temporal overlay, evidence-based practice, assessment, child welfare, child abuse, child maltreatment, families, depression, and homelessness had earlier average publication years. COVID-19, stress, adverse childhood experiences, resilience, social support, social work practice, youth, and substance use had more recent average publication years. Average publication year describes the relative temporal position of a keyword within the mapped corpus; it does not, by itself, demonstrate the emergence, decline, replacement, or causal development of a topic.

## 4 Discussion

### 4.1 Growth and interdisciplinary expansion

The corpus grew steadily over 26 years, with particularly rapid expansion after 2017. The timing aligns with increasing global concern about child and adolescent mental health, the effects of the COVID-19 pandemic, and calls to reorganize services around early intervention and community access (1–5, 29). The 8.3% growth rate establishes the scale of expansion, while its distribution across professional and service domains carries the greater disciplinary significance. Social work journals formed a recognizable base, yet much of the influential scholarship appeared across child welfare, psychiatry, psychology, pediatrics, public health, and implementation research. Expansion thus occurred through interdisciplinary connection rather than through consolidation as a self-contained specialty. This increase concerns indexed publication output and does not establish a parallel expansion of services, greater influence of social work in practice, or improvement in the methodological quality of the underlying studies.

The growth and disciplinary dispersion observed in this corpus are consistent with previous bibliometric findings, although the analytical scopes differ. Chen et al. reported increasing publication output, expanding interdisciplinary collaboration, and Western concentration in child and adolescent mental health intervention research (16). Pandemic-focused mappings likewise documented rapid growth and broad disciplinary participation in child and adolescent mental health research (17), while Duru identified connections between social work journals and public health, medicine, and psychiatry in the COVID-19 literature (19). The present findings indicate that growth and disciplinary dispersion are not confined to intervention research or the pandemic period; they are also visible across a 26-year corpus organized around social work and child and adolescent mental health. The additional contribution lies in showing how this dispersion connects social work with child welfare, schools, family services, pediatric settings, and community care within the same knowledge structure.

The term “social work scholarship in child and adolescent mental health” refers to the operationally bounded corpus defined by the search and eligibility criteria. The corpus includes publications explicitly addressing psychiatric, mental health, or clinical social work as well as records from adjacent service domains that demonstrate a substantive connection among social work, a child or adolescent population, and mental health. The term therefore describes the scope of the eligible literature rather than a formally bounded specialty or a closed inventory of psychiatric social work.

The dispersed source structure suggests that the field lacks a single publication home. Although such dispersion supports cross-disciplinary exchange, it can make cumulative knowledge harder to identify. Pediatric clinicians may encounter work on family stress and social determinants in one literature, child welfare researchers may find service-use studies in another, and social work researchers may publish implementation studies outside clinical journals. Bibliometric mapping makes those relationships visible while leaving the underlying fragmentation unresolved.

### 4.2 Convergence around cross-sector care

The co-citation and keyword maps describe different layers of the same knowledge structure. Cited-author clusters identify the traditions on which the field draws, whereas keyword clusters show the problems, populations, and service settings examined in the publications. Developmental and ecological scholarship aligned with family and social-determinant terms; implementation research corresponded to service-system, child-welfare, and school-social-work terms; trauma and resilience scholarship converged with adversity and child-protection themes; and practice and workforce traditions were reflected in clinical work and organizational conditions. Across both maps, child and adolescent mental health appeared as a service-system concern as well as a clinical one.

The convergence accords with contemporary service guidance. WHO and UNICEF call for community-based networks that connect health, education, social care, and juvenile justice while involving children and families in service design (1). Integrated youth mental health models show potential to improve access, experience, and outcomes, although the evidence remains heterogeneous (13). Research on integrated health and social care for underserved children likewise identifies promising models but notes weak evaluation, organizational barriers, and limited recognition of social care roles (14). Child welfare, school social work, family support, and trauma-informed care provide the clearest links between the mapped literature and this policy agenda.

The visibility of child welfare, child protection, maltreatment, trauma, and foster care is consistent with previous mappings of adjacent research domains. Tran et al. identified child maltreatment as a large, internationally distributed, and interdisciplinary research field (18). More directly, Cebeci mapped child abuse and neglect through a social work lens and identified child welfare and child protection as central elements of the field's thematic development (20). The present analysis confirms the importance of these domains but places them within a wider child and adolescent mental health structure. In this broader corpus, child welfare and protection were connected with school social work, family practice, implementation research, youth mental health treatment, and community support. The contribution is therefore not the first identification of child welfare within social work-oriented bibliometric research, but the mapping of its relationships with other mental health and service-system domains.

Child welfare occupied a central position across sources, keywords, and cited traditions. This pattern is consistent with evidence concerning adversity, mental health problems, and multi-setting service use among children involved with protective systems (15, 30, 31). The observed co-citation and keyword relations identify a recurring scholarly interface between child protection and mental health services. They do not establish which arrangements for shared assessment, coordinated care planning, family engagement, or interagency responsibility are effective. The comparative effectiveness and implementation of such arrangements require direct evaluation.

School social work and school mental health formed another important strand. Schools offer access to children who may never reach specialist services and provide a setting in which emotional distress, peer relationships, learning, family concerns, and safeguarding can be considered together (10, 11). The visibility of school social work and school mental health is consistent with a preventive and community-oriented strand of scholarship. School mental health was positioned as a specialized theme in the thematic map rather than as a basic or motor theme. This position describes its relative centrality and density within the selected corpus and thresholds; it does not establish that school-based services are operationally separated from pediatric or mental health systems.

### 4.3 Trauma, resilience, and continuing clinical concerns

Trauma, adverse childhood experiences, resilience, and social support became more visible in recent periods. The development is consistent with evidence linking childhood adversity and maltreatment to long-term mental health outcomes and with the spread of trauma-informed approaches across child-serving systems (30–34). The co-citation and keyword patterns placed trauma-related scholarship near child welfare, family practice, community support, and implementation terms. This proximity represents conceptual association within the indexed corpus and does not demonstrate that trauma-informed approaches were implemented consistently or produced effective service outcomes.

The findings do not support a simple narrative in which the field moved from pathology to resilience. Depression, anxiety, suicide, substance use, treatment, and psychiatry remained visible. The change was additive: clinical concerns were increasingly situated alongside adversity, family context, social support, and service organization. This is a more plausible account of disciplinary development than claims of a complete paradigm shift.

The resilience language also requires care. Resilience can direct attention to strengths, adaptation, and protective relationships, but it can become individualizing when structural barriers are ignored. In the mapped literature, the proximity of resilience to social support, homelessness, refugees, child welfare, and community terms offers some protection against that reduction. Future research should continue to examine whether resilience-oriented interventions alter social conditions or merely ask children and families to adapt to them.

### 4.4 Geographic concentration and transferability

Country participation was concentrated in the United States, United Kingdom, Canada, and Australia, although the full-counting country shares are non-exclusive and cannot be summed. This pattern is broadly consistent with previous bibliometric studies. Chen et al. reported Western concentration in child and adolescent mental health intervention research (16), Duru identified the United States as the leading contributor to social work scholarship during COVID-19 (19), and Cebeci found that social-work-oriented child abuse and neglect research was concentrated in high-income countries with the United States occupying a central collaboration position (20). The present analysis shows that a similar geographic pattern is visible across the wider intersection of social work and child and adolescent mental health. English-language eligibility and the coverage of Web of Science and Scopus are plausible contributors to this distribution, but the bibliometric data cannot determine the relative effects of database coverage, research capacity, funding, or differences in national service infrastructures.

Transferability is constrained because social work roles depend on welfare arrangements, workforce regulation, professional recognition, family policy, and the availability of community services. Models developed in well-resourced systems cannot be applied directly where specialist child mental health care is scarce, social workers hold different mandates, or families rely mainly on informal and community supports. WHO and UNICEF report particularly limited child and youth mental health resources in low- and middle-income countries and recommend locally adapted models (1). Such settings remain disproportionately underrepresented in the indexed knowledge base.

International collaboration was visible but relatively limited: fewer than one in six records with country data involved multiple countries. Cross-national work is important not only for increasing publication counts but for testing whether concepts and service models retain meaning across cultural and institutional contexts. Partnerships should move beyond data extraction from underrepresented settings. Co-designed research, equitable authorship, local leadership, and attention to non-English scholarship are necessary if the field is to claim global relevance.

### 4.5 Pediatric practice and service-organization research priorities

The bibliometric findings establish patterns of scholarly visibility, co-citation, keyword co-occurrence, and thematic development. They do not evaluate service performance, intervention effectiveness, policy implementation, or the outcomes of specific social work roles. Accordingly, the implications outlined here are framed as priorities for empirical investigation rather than as conclusions about how pediatric or mental health services currently operate.

Family support, trauma, child protection, school social work, assessment, collaboration, and service-system terms were visible across the mapped structures. These patterns identify interfaces at which social work contributions could be examined in pediatric settings. Evaluative studies could test whether and under what conditions social work involvement in psychosocial assessment, screening pathways, referral, care coordination, family support, safeguarding, and community linkage is associated with access, continuity, family experience, equity, or functional outcomes. Comparative, implementation, and longitudinal designs are needed before conclusions can be drawn about the effectiveness of these roles or service arrangements.

Primary care, integrated pediatric care, digital mental health, telehealth, and formal health–social care integration were comparatively less visible among keywords meeting the selected occurrence threshold. This finding identifies a gap in the high-frequency indexed literature, not an absence of these services in practice. Research could therefore examine how social work is incorporated into pediatric primary care and integrated behavioral health models, which activities are assigned to social workers, and whether these arrangements influence access, waiting-period support, continuity across services, and family outcomes.

Policy implications are similarly provisional. Collaboration, implementation, child welfare, school social work, and workforce strain were visible in the bibliometric structures, but the maps cannot determine whether particular referral pathways, information-sharing arrangements, financing mechanisms, staffing models, or training programs are preferable. Policy and implementation research could compare these arrangements across service systems and welfare contexts. Relevant outcomes may include referral completion, continuity of care, service accessibility, family participation, workforce well-being, implementation fidelity, and health equity.

Additional priorities include culturally responsive research in low- and middle-income settings, comparative evaluations across different welfare systems, digital and hybrid approaches that do not widen exclusion, child and family participation in service design, and implementation studies that specify social work activities and organizational conditions. Bibliometric mapping identifies where research is concentrated and where visibility is limited; primary evaluative research is required to determine which models merit adoption or wider implementation.

### 4.6 Strengths and limitations

The study combined Web of Science and Scopus records using documented search, integration, and deduplication procedures. Performance analysis, co-citation, keyword co-occurrence, thematic mapping, and thematic evolution were used to examine temporal, intellectual, conceptual, and geographic patterns within the operationally defined corpus.

Coverage was restricted to English-language journal articles and reviews indexed in Web of Science or Scopus between 2000 and 2025. PubMed/MEDLINE, CINAHL, PsycINFO, and regional databases were not searched, while books, book chapters, dissertations, practice reports, policy documents, and gray literature were outside the eligibility criteria. These decisions may have reduced the visibility of pediatric, nursing, psychological, practice-oriented, regional, and non-English scholarship. They may also have contributed to the observed geographic and source concentration. The corpus should therefore be understood as a structured representation of the literature captured by the specified databases and eligibility criteria rather than as a complete representation of global knowledge production.

The search required explicit social work terminology in the bibliographic metadata. This requirement increased conceptual specificity but may have excluded relevant studies that described social work roles using different professional or service terminology. Conversely, the inclusion of adjacent settings such as child welfare, schools, foster care, and integrated services created the possibility of including records with only peripheral social work relevance. Relevance screening was conducted by a single reviewer at the metadata level and was not independently duplicated. Although explicit decision rules and re-examination of ambiguous records were used to improve consistency, classification at the boundary between broad child mental health research and substantively social-work-oriented scholarship necessarily involved judgment. The absence of independent agreement data limits the auditability of these decisions and means that some boundary records may have been misclassified.

Differences in metadata supplied by Web of Science and Scopus may affect author names, affiliations, country identification, keywords, cited references, and citation counts. Field-level reconciliation reduced but could not eliminate these inconsistencies. Keyword harmonization also required semantic judgment; alternative decisions about merging or retaining terms could produce different co-occurrence structures. Cluster labels were developed by the author and remain interpretive despite review of complete cluster membership and prominent nodes. Network thresholds improved visual readability but excluded low-frequency items, including potentially important emerging topics. Because no formal threshold-sensitivity analysis was conducted, the number, composition, and relative prominence of smaller clusters could differ under alternative cutoffs. Absence from a thresholded map should therefore be interpreted as lower visibility under the selected parameters rather than absence from the corpus.

Citation and co-citation indicators are influenced by publication age, database coverage, and disciplinary citation practices. Recent publications have had less time to accumulate citations and may appear less prominent even when they address developing areas. Bibliometric analysis does not assess study quality, intervention effectiveness, service performance, or policy impact. The thematic and disciplinary interpretations are therefore bounded by the indexed corpus, the available metadata, and the selected analytical parameters. Focused systematic reviews, qualitative syntheses, implementation studies, and outcome evaluations are required to assess the quality and practical implications of the mapped scholarship.

## 5 Conclusion

Within the English-language journal articles and reviews indexed in Web of Science and Scopus between 2000 and 2025, publication output increased but remained geographically concentrated. Co-citation and keyword maps showed multiple intellectual traditions and the recurring visibility of child welfare, family and school practice, trauma, implementation, and youth mental health. Mental health and social work showed thematic continuity, whereas integrated pediatric and primary care were less visible among keywords meeting the selected occurrence threshold.

These patterns describe the indexed corpus and do not establish research quality, service effectiveness, or current service organization. Continued knowledge development should be accompanied by evaluative, comparative, and implementation-oriented research on social work contributions to access, coordination, continuity, family experience, and equity across pediatric mental health and related service systems.

## Data Availability

The original contributions presented in the study are included in the article/Supplementary Material, further inquiries can be directed to the corresponding author.

## Supplementary material

SUPPLEMENTARY TABLE S1 Representative keyword harmonization decisions.
