# Supplementary material for: Mapping social work scholarship in child and adolescent mental health: a bibliometric analysis of Web of Science and Scopus, 2000–2025
Source: Front Pediatr. 2026 Sep 10;14:1936049. doi: 10.3389/fped.2026.1936049 (PMC13601354; doi:10.3389/fped.2026.1936049)
Supplement: Supplementary file 1 [file Table1.docx]

**Supplementary Table S1. Representative keyword harmonization decisions**

| **Decision** | **Original term(s)** | **Term used in the analysis** |
| --- | --- | --- |
| Merged | children; child | child |
| Merged | adolescents; adolescent | adolescent |
| Merged | social workers; social worker | social worker |
| Merged | paediatric; pediatric | pediatric |
| Merged | behavioural health; behavioral health | behavioral health |
| Merged | PTSD; posttraumatic stress disorder; post-traumatic stress disorder | post-traumatic stress disorder |
| Merged | COVID 19; COVID-19; coronavirus disease 2019 | COVID-19 |
| Merged | case-management; case management | case management |
| Retained separately | child welfare; child protection | child welfare; child protection |
| Retained separately | child abuse; child maltreatment | child abuse; child maltreatment |
| Retained separately | trauma; adverse childhood experiences | trauma; adverse childhood experiences |
| Retained separately | social work; social services | social work; social services |
| Retained separately | mental health; psychiatry | mental health; psychiatry |
| Retained separately | family support; social support | family support; social support |
| Retained separately | child; adolescent; youth; young people | child; adolescent; youth; young people |
| Retained separately | clinical social work; psychiatric social work; school social work | clinical social work; psychiatric social work; school social work |

Note: Terms were merged only when they represented lexical variants, spelling variants, abbreviations, or semantically equivalent expressions. Conceptually related terms were retained separately when their distinction was relevant to population, service setting, professional role, or thematic interpretation.
